# The Most Active Child Is Not Always the Fittest: Physical Activity and Fitness Are Weakly Correlated

**DOI:** 10.3390/sports11010003

**Published:** 2022-12-22

**Authors:** Corrado Lupo, Paolo De Pasquale, Gennaro Boccia, Alexandru Nicolae Ungureanu, Paolo Moisè, Anna Mulasso, Paolo Riccardo Brustio

**Affiliations:** 1 NeuroMuscularFunction, Research Group, School of Exercise & Sport Sciences, University of Turin, 10126 Turin, Italy; 2 Department of Medical Sciences, University of Turin, 10126 Turin, Italy; 3 Department of Clinical and Biological Sciences, University of Turin, 10126 Turin, Italy; 4 School of Exercise & Sport Sciences, University of Turin, 10126 Turin, Italy

**Keywords:** PAQ-C, physical tests, active children, non-active children, sedentary, physical skills, coordinative capabilities, conditional capabilities

## Abstract

The present cross-sectional study aimed to evaluate the impact of physical activity level (PA) on physical fitness by controlling for individual characteristics in Italian children. A total of 329 children (girls *n* = 155, 42.6%; from five primary schools, 17 classes) aged 8–10 filled out the Physical Activity Questionnaire for Older Children (PAQ-C) to assess their PA level and performed anthropometric measurements (body mass, height, and BMI) and physical tests for measuring sprint (20 m sprint), cardiorespiratory fitness (shuttle-run test), balance (single-leg stance), handgrip strength (handgrip), lower-limb power (standing long-jump), peak force (countermovement jump), and low-back flexibility (sit-and-reach) skills. Linear mixed-effects models were applied to determine the relationship between physical fitness and PAQ-C score controlling for individual characteristics (i.e., gender, age, BMI). Results reported significant relationships between PAQ-C scores and sit-and-reach, shuttle-run, long-jump, and sprint tests. All considered physical tests were correlated with gender, age, and BMI, except for sit-and-reach from BMI. The variance in age, gender, BMI, and PAQ-C score accounted altogether for 30.0% of the variance in handgrip, 23.0% in single-leg stance, 26% in sit-and-reach, 36% in shuttle-run, 31% in long-jump, 34% in sprint, and 31% in countermovement jump. Therefore, the relationship between PA and fitness is not absolute and depends on the test and children’s characteristics.

## 1. Introduction

Regular physical activity (PA) is the most crucial action that people of all ages can consider to improve their health. Moving more and sitting less powerfully benefit everyone regardless of age, sex, or fitness [1]. Specific considerations and health-related recommendations need to be provided for children and adolescents. International guidelines recommended that children and adolescents aged 5–17 years spend at least an average of 60 min per day doing physical activity at moderate-to-vigorous intensity [2], or several hours of a variety of structured and unstructured light PA across the week [3], to limit the amount of time spent being sedentary, and particularly the amount of recreational screen time [2]. Despite PA’s widespread positive health benefits, most children and adolescents do not reach these international recommendations [4]. For instance, in Italy, only one child aged 6–9 years practices sufficient PA (i.e., at least 1 h a day), while one child over four watches television for 4 h or more daily [5]. Reaching the recommended PA level is positively associated with augmented physical and psychological health [6,7,8] and social factors [9,10,11]. For example, a higher level of PA can reduce body fat and cardiovascular and metabolic disease risk profiles [1]. Additionally, during childhood, the daily PA may positively impact the development of fundamental movement skills, the acquisition of motor skill competence, and movement confidence [12,13], as well as the enhancement of fitness (defined as the attributes related to a person’s ability to perform specific physical tests [14]). Interestingly, a physically active lifestyle during childhood and adolescence is robust and tracks into adulthood [12]. Clearly, a better understanding of the association between PA and fitness in childhood may have practical implications for planning actions for health enhancement, especially considering that PA is a modifiable lifestyle behavior [15].

In this regard, the Physical Activity Questionnaire for Older Children (PAQ-C) is one of the most promising self-reported measures to discern general levels of PA over the last seven days in children aged 8–14 [16]. Due to its practicality, it may be considered a practical tool used in population-based studies in the school context [16]. However, controversial results exist about the relationship between PA (i.e., PAQ-C score) and fitness. Thomas et al. [17], evaluating children aged 9–11 from the UK, found a moderate inverse correlation between PAQ-C score and ½ mile walk run test (r =−0.38), but not with body mass index (BMI). In Japanese children aged 9–12, the PAQ-C was significantly associated with athletic competence (r = 0.41), self-efficacy (r = 0.65), body fat percentage (r = −0.19), and the YMCA 3-Minute Step Test (r = −0.32), but it was not significantly correlated with BMI (r = −0.09) [18]. Similarly, the Canadian Home Fitness Test (i.e., step test) was weak (r = 0.28) related to PAQ-C [19]. More interestingly, the PAQ-C was not correlated with another step test (i.e., a modified version of the Harvard step test) or BMI in African American or Hispanic children but showed weak-to-moderate relationships with the BMI (r = −0.16) and cardiovascular fitness (r = 0.30) in European American children [20]. Nevertheless, a higher relationship was found in older children (aged 10–14), where PAQ-C explained about 36% of the variance in VO_2max_ evaluated with the shuttle-run test [15]. Interestingly, the PA level assessed with PAQ-C influenced gross motor coordination (estimate B = 0.52) when controlling for age, lean body mass, and height [21]. Differently, PAQ-C results positively correlated with the maximal exercise capacity (r = 0.55) and negatively associated with BMI (r = −0.79) in children with less severe congenital heart disease but not with children with a more severe one [22].

Summing up, the concurrent evidence underlined as the PAQ-C demonstrated weak-to-moderate convergence of fitness underlined as the cultural context may affect the results. Moreover, several other factors (e.g., genetics, skills, coordination, and motivation for PA), regardless of practiced PA, could substantially determine different outcomes on fitness. To the best of our knowledge, only one study on healthy Italian children investigated this issue with mixed results, especially concerning gender. In this study, Sacchetti et al. [23] found that daily PA, quantified using a modified PAQ-C, was positively associated with boys’ explosive upper-limb strength, sprint, and flexibility, whereas only flexibility correlated for girls (aged 8–9). It is still unclear if a satisfactory practice of PA assessed with the PAQ-C is always correlated to fitness regarding the main conditional (i.e., upper-limb strength and lower-limb power, cardiorespiratory fitness, sprint) and coordinative (i.e., balance, flexibility) capabilities, especially considering the considerable heterogeneity of children [24,25].

For this reason, the replication of fitness tests considered in previous studies on the same nationality (i.e., sit-and-reach for low-back flexibility; standing long-jump for lower-limb power; and 20 m running sprint) [23] and age (i.e., handgrip strength for upper-limb strength [26]; single-leg stance test for balance [27]; shuttle-run for cardiorespiratory fitness [26]) categories of children should be encouraged together with other measurements characterized by the application of less traditional instruments and modalities (e.g., the countermovement jumps on the force platform, in addition to the standing long-jump test, to measure the lower-limb peak force and power, respectively). Thus, this cross-sectional study aimed to evaluate the relationship between PA level (assessed through the PAQ-C) and fitness skills (including handgrip test for upper-limb strength; single-leg stance for balance; sit-and-reach test for low-back flexibility; 20 m shuttle-run for cardiorespiratory fitness; standing long-jump test and countermovement jumps for lower-limb power and peak force, respectively; and 20 m linear-sprint test for sprint ability), by controlling for individual characteristics (i.e., age, gender, BMI) in Italian children aged 8–10 years.

## 2. Materials and Methods

### 2.1. Participants

Sessions to provide preliminary information regarding the aim of the study to the children’s teachers and parents/guardians were held in five primary schools (17 classes in total) in Turin neighborhoods (Northwest Italy). All schools/classes recruited for the study were part of the same institutional and regional office and, therefore, were receiving a similar delivery of educational curricula [28,29,30]. According to the ethical standards in the Declaration of Helsinki, children, parents/guardians, and teachers provided written informed consent for the study. Before data collection, the institutional review board of the University of Turin approved this study (Protocol #134691; 9 March 2020). Three-hundred-and-sixty-four children and relative parents/guardians gave their written informed consent after receiving detailed information about the aims and procedures of the study. Absent subjects on the day of data collection (*n* = 36) were excluded. A final sample of 329 children (girls *n* = 155, 42.6%) aged 8–10 participated in this study.

### 2.2. Procedures

In two weekly classes, all measurements were performed between 13 April and 10 June 2021. One class was tested between 8.30 and 10.30 a.m., and the other between 10.30 and 12.30 a.m. At the beginning of each testing session, classes were split into two subgroups. One subgroup filled the PAQ-C while, in the meantime, the other subgroup performed the anthropometric measurements and physical tests. Then, the two subgroups were inverted. In a randomized order, data about physical and anthropometric measurements were collected in small groups (i.e., 5/6 children each, depending on the class size). All the children performed the cardiorespiratory fitness evaluation at the end of the measurement session. Test sessions were organized in school gyms with similar superficies and space to guarantee equal test circumstances and satisfactory safety measures. The same qualified investigators performed all measurements using a standardized test protocol. During the test session, participants were encouraged to provide the best fitness in each physical test.

#### 2.2.1. Physical Activity Questionnaire for Older Children (PAQ-C)

Self-reported PA was assessed using the Italian version of the Physical Activity Questionnaire for Older Children (PAQ-C) [22], consisting of ten items (despite the tenth item not being considered for the final summary score) [19]. The questionnaire collects information on participation in different types of activity, sports, and efforts during physical education classes, during lunch, after school, in the evening, and during the weekend, within the past seven days [19].

The first item is a checklist of activities and common sports and games. The following six items assess PA during specific moments of the previous seven days: physical education classes, breaks, lunchtime, right after school, in the evening, and during the weekend. The eighth items evaluate PA practiced during free time, and the ninth item asks about the frequency of PA in general over the previous week. Out of the total score calculation, the tenth item aims to ask children if they are sick or if something precludes them from doing their regular PA. For each item, a score between 1 (low PA) and 5 (very high PA) could be provided by the single participant, and the average score denotes the final PAQ-C activity summary score; therefore, high scores indicate higher levels of PA. Although the Italian version of the PAQ-C differs from the original English version, especially for the activities reported in the first item, it is acceptable to have good internal consistency (Cronbach’s alpha values 0.70–0.83) [22].

#### 2.2.2. Anthropometric Parameters

Stature was measured using a portable stadiometer (Model 214; Seca, Hamburg, Germany) with an accuracy of 0.01 m. Body mass was measured using an electronic scale (Model 876; Seca, Hamburg, Germany) with an accuracy of 0.1 kg. Both stature and body mass were measured without shoes. The Body Mass Index (BMI) was calculated as the body mass divided by height squared (kg m^−2^).

#### 2.2.3. Handgrip Strength

Handgrip strength was measured using a manual dynamometer (Baseline Smedley digital hand dynamometer, model 12-0286). During the test, participants stood with their dominant arms constantly vertical and close to the body. The palm did not flex on the wrist joint. The subjects were required to exert maximal strength on the dynamometer (maximum voluntary contraction) once using the dominant hand. The dynamometer scale indicated handgrip strength in kilograms (with an accuracy of 0.1 kg).

#### 2.2.4. Balance

Balance was tested using the single-leg stance test. The dominant leg of the participants was determined by asking them which was their favorite leg [27]. Children stand on one leg with the other knee flexed, hands on the hips, for a maximum of 120 s, looking straight ahead. Subjects performed all tests without shoes. The subject could not move the foot from the initial position during the test. Compensatory movements of the body and lifted leg were allowed but not of the arms. Children performed the test in subgroups of three participants. Time was individually stopped when the child moved from the mark or put the foot of the flexed leg down. The time (in seconds) was recorded using a stopwatch with an accuracy of 0.01 s.

#### 2.2.5. Low-Back Flexibility

The sit-and-reach test was applied to assess hip and low-back flexibility. The child was seated on the floor with knees fully extended and ankles in neutral dorsiflexion against the sit-and-reach box (Standard Flexibility Tester, Baseline, New York, NY, USA). The child was instructed to place one hand on the other and slowly reach forward as far as possible while keeping the knees extended. The hands were kept aligned evenly as the subject reached forward along the box’s surface. The final position of the fingertips on the ruler was recorded (expressed in cm) [31]. Each child practiced the test twice; the best result was considered for further statical analysis.

#### 2.2.6. Cardiorespiratory Fitness

The 20 m shuttle-run test, according to the Leger test soundtrack version (CAEP Quebec Faca), was used to evaluate the cardiorespiratory fitness. The test was performed once and consisted of running in small groups back and forth (i.e., in shuttles) between two lines 20 m apart, following signals played from a pre-recorded soundtrack (beep). The test started at a speed of 8.5 km h^−1^ and increased by 0.5 km h^−1^ every minute according to audio signals emitted at set intervals from the Leger pre-recorded soundtrack [32]. The end of the test occurs when the child stops on his/her own to fatigue or when he/she does not reach the line in time (i.e., before the correspondent audio signal) on two consecutive occasions [26]. The test score was the last reached stage (converted into the corresponding distance expressed in meters) [26]. Subgroups of six children executed the test, supervised by a minimum of four investigators to monitor test performances and establish the end of the test for each participant.

#### 2.2.7. Lower-Limb Muscle Power

The standing long-jump test was performed to assess lower-limb power. Children jumped as far as possible off the stand, trying to land with both feet together and maintaining equilibrium once they landed (it was not allowed for them to put their hands on the floor, and the closest foot to the take-off line was considered in case of asymmetric landing feet). The test measures the distance jumped (i.e., the distance between the last heel mark and the take-off line) using a measurement to the nearest 0.01 m. Two trials were performed (with a 1 min pause in between), and the best score was considered.

#### 2.2.8. Lower-Limb Peak Force

In addition to the standing long-jump test, after a short briefing and one test trial, each child performed two countermovement jumps on a force platform (C-Force Performance Platform System, Innervations, West Perth, WA, Australia), with a rest between trials of around 3 min. The force platform signal was sampled at 1000 Hz and filtered using a low-pass filter with a cutoff frequency of 50 Hz. Each participant started in a standing position on the force platform with their knees extended and keeping their arms akimbo. Children performed the jumps as quickly and high as possible. Peak vertical force (Peak-force-CMJ) was defined as the maximal force recorded in the two trials expressed according to the ground reaction force (minus body weight) data [33], measured to the nearest 0.01 N.

#### 2.2.9. Sprint Ability

Children’s sprint ability was evaluated by the 20 m linear sprint test [34]. Individually, participants started the test with feet behind the startling line [35] and ran the 20 m distance as fast as possible, twice with a 1 min rest between the trials. The time elapsed from the start to the finish line was measured through infrared reflex photoelectric cells (Witty—Wireless Training Timer; Microgate, Udine, Italy), measuring to the nearest 0.01 s.

### 2.3. Statistical Analysis

Descriptive data (means and 95% CI) of the subjects’ characteristics (i.e., age and BMI), PAQ-C scores, and physical tests (i.e., handgrip, single-leg stance, sit-and-reach, shuttle-run, lower-limb power, 20 m sprint, and Peak-force-CMJ) were reported for the whole sample with consideration of gender.

Pre-processing involved detecting and removing outliers using the Cook distance methodology [36]. Linear mixed-effects models (LMM) were applied to determine the relationship between fitness and PAQ-C score controlling for individual characteristics (i.e., gender, age, BMI). Fitness (i.e., handgrip, single-leg stance, sit-and-reach, shuttle-run, long-jump, 20 m sprint, and Peak-force-CMJ) were used as the dependent variable, and individual characteristics (i.e., age, gender, BMI) and PA level (i.e., PAQ-C) as independent factors. In all LMM, classes and schools were considered nested random effects (i.e., class in school) to account for errors for repeated measures. R^2^ and partial R^2^ using implementation parametric bootstrapping better describes this relationship between the predictor and the independent variables. The significance level was set at 5% (*p* < 0.05). All data were analyzed using statistical package R (version 4.0.3; Foundation for Statistical Computing, 2018) with the packages “lme4” [37], “emmeans” [38], and “partR2” [39]. Force platform analysis was performed using custom-written software in MATLABR2022b (MathWorks, Natick, MA, USA).

## 3. Results

Table 1 reports descriptive data (means and 95% CI) of the subjects’ characteristics, physical activity (i.e., PAQ-C scores), and fitness tests.

Table 2 presents the results of the LMM analysis. The variance in PAC-Q, Gender, Age, and BMI accounted altogether for 30% of the variance in handgrip strength (R^2^ = 0.30, [0.23, 0.40]), 23.0% in single-leg stance (R^2^ = 0.23, [0.15, 0.31]), 26% in sit-and-reach (R^2^ = 0.26 [0.15, 0.32], 36% in shuttle-run (R^2^ = 0.36, [0.29, 0.41]), 31% in long-jump (R^2^ = 0.31, [0.27, 0.39]), 34% in sprint test (R^2^ = 0.34, [0.29, 0.42]), and 31% in peak force CMJ (R^2^ = 0.31, [0.25, 0.39]). In particular, sit-and-reach, shuttle-run, long-jump, and sprint tests significantly correlated to the PAQ-C (Partial R^2^ ranged = 0.08–0.14). Generally, better fitness results positively correlated with higher PAQ-C scores. The only negative direction emerged for the sprint test (i.e., lower performances corresponding to more extended time in the test). Differences between males and females emerged in all considered physical tests (Partial R^2^ ranged = 0.05–0.23). Boys reported better fitness in handgrip strength, shuttle-run, long-jump, and sprint test. Girls showed better fitness for single-leg stance, sit-and-reach, and Peak-force-CMJ than boys. Concerning age, significance emerged for the results of all physical tests (Partial R^2^ ranged = 0.09–0.17). In particular, older children reported higher fitness levels than younger ones. Finally, considering BMI, significance emerged for the results of all physical tests (Partial R^2^ ranged = 0.09–0.17) except for sit-and-reach results. A higher BMI was negatively associated with single-leg stance, shuttle-run, long-jump, and sprint performance, but was positively associated with handgrip strength and Peak-force-CMJ. More details about unstandardized regression coefficients (standard error) and Partial R^2^ are available in Table 2.

## 4. Discussion

The present cross-sectional study aimed to evaluate the associations between PA level, measured according to the PAQ-C score, and some fitness tests regarding cardiorespiratory fitness, handgrip strength, lower-limb power, peak force, sprint, balance, and low-back flexibility skills, by controlling for individual characteristics (i.e., age, gender, BMI), in Italian children aged 8–10. The main finding was that PA (i.e., PAQ-C score) was weakly associated with the considered fitness tests when controlling for gender, age, and BMI. Data suggested only a weak (but significant) relationship with cardiorespiratory fitness (i.e., shuttle-run test), lower-limb power (i.e., long-jump test), sprint (i.e., 20 m sprint test), and low-back flexibility (i.e., sit-and-reach). In contrast, no relationship between PAQ-C scores and handgrip strength or peak force in the CMJ test emerged.

According to Sacchetti et al. [23], PA improves children’s fitness, notably for boys’ explosive upper-limb strength, 20 m running speed, strength, and low-back flexibility (the latter skill in girls too). Similarly, we found a negative relationship between the PAQ-C score and sprint (actually positive in performance and better for boys) and positive for low-back flexibility (better for girls). Conversely, in our results, no effect emerged for handgrip strength (despite being measured through a handgrip dynamometer instead of a medicine-ball throw); the standing-long-jump was affected by PA level only in our results, despite no effect emerging for CMJ peak force. For the latter results, it is possible to hypothesize that children reported a heterogeneous incapacity of coordinatively maximizing their jump skills on a plate force [40]. Finally, likely for lower-limb peak force, the single-leg stance test is not characterized by stable results with high heterogeneity and somatotype difference [41], even if exclusively performed with eyes opened. Therefore, the association between children’s PA levels and fitness seems complex and only partially confirmed [23].

Moreover, it is necessary to point out that the associations between the PAQ-C and cardiorespiratory fitness, lower-limb power, sprint, and low-back flexibility tests are weak despite their significance. Indeed, the partial R^2^ ranged from 0.08 to 0.14 (Table 2). Therefore, data corroborates that PAQ-C scores and fitness in childhood showed weak-to-moderate relationships. For example, focusing on cardiorespiratory fitness, the final model explained 36% of the variance, and PAQ-C only 8%. Similarly, age, gender, BMI, and PAQ-C accounted for 26% of low-back flexibility, 31% of lower-limb peak force, and 34% of sprint ability, whereas PAQ-C explained only 14%, 13%, and 10%, respectively. On the other hand, it is important to note that other factors will account for the remaining variance. For instance, genetic factors may be a key element in these associations [42]. In addition, it is possible to also speculate that factors such as physical self-concept, motivation to PA, and gross motor coordination may contribute to this association. Thus, considering that the PAQ-C can explain a lower percentage of variance associated with the scores of the observed fitness tests, it is possible to suggest that children with higher levels of PA are not necessarily fitter.

Regarding children’s gender, age, and BMI, the present results aligned with the literature [26]. Boys generally performed better than girls in sprint [23], handgrip strength [26], cardiorespiratory fitness [26], and lower-limb power. Differently, girls showed more competence in balance [23] and low-back flexibility [26] skills. Surprisingly, peak force measured during CMJ resulted better in girls than boys. It is possible to speculate that the early maturation of girls may specifically influence muscular peak force (and not power). This factor might increase the ability to perform more successfully than boys. Indeed, in our study, we considered only chronological age and not maturity, which may affect both self-reporting PA and fitness measures, especially for girls, who generally reach maturation before boys [43]. Finally, gender differences may also be explained by motivational aspects, social interest, or peer influence influencing the PA attendance and type of sport practiced [44].

Overall, older children performed better on physical tests than younger children, confirming the same trend that emerged from previous studies for handgrip, balance (through the flamingo test in that case), and long-jump test [23]. Nevertheless, as already reported, we considered only chronological age and not maturity, which may affect both self-reporting PA and physical test measures, especially for girls, who generally reach maturation before boys [43].

Except for low-back flexibility, our results reported that children with a higher BMI are stronger in handgrip than children with lower values. However, they showed lower levels both in cardiorespiratory fitness and sprint skills, as well as in single-leg stance and long-jump tests, thus confirming data on children of the same age and nationality [23], European American [20], and with less severe congenital heart disease [22].

Counterintuitively, especially considering the reverse relationship between children’s BMI and long-jump performances, children with higher BMI reported better fitness in Peak-force-CMJ, hypothesizing that the execution of the latter test is potentially characterized by lower execution difficulties than the first one, for so young a subject category. Therefore, even though children were instructed about how to perform the jumps on the force plate, the two tests were possibly affected by a different familiarization process, thus providing a relevant personal limitation to the truthfulness of results. As an additional limit, the high number of tested children and their young age constricted the investigators to follow a specific subgroup of children and be interchangeable among test stations, thus potentially affecting results according to their grade of operator dependency. Moreover, the PA of the past week could be hard to remember for these participants. For these reasons, we supported and encouraged children to provide their best fitness in each physical test; nevertheless, it is necessary to point out that children’s motivation may mitigate the results during the test session.

## 5. Limitations

In addition to the already mentioned experimental limitations (i.e., a self-reported quality measurement to assess PA level; different familiarization processes in executing tests; different operator-dependency; children’s difficulty in remembering PA level in the last week), this study is also characterized by further potential biases. Due to the cross-sectional nature of our study, a causal relationship between the physical fitness test and the PA level cannot be drawn. Additionally, only some physical measures were assessed, and some are not representative of the entire physical conditioning (e.g., shuttle-run can be considered as a test for cardiorespiratory fitness but not for endurance in general; seat-and-reach assesses low-back flexibility and not global flexibility). Finally, other measurements (e.g., abdominal strength, coordination) associable with a fitness evaluation are not considered.

For these reasons, future longitudinal research could be provided on a wider series of fitness tests, also evaluating other youth categories (e.g., older children), and simultaneously promoting physical activity interventions such as physical activity led and structured by a physical activity and sport science expert (i.e., in line with evidence-based directions), or physical activity led by the school teaching staff, according to brief daily outdoor activities (e.g., The Daily Mile).

## 6. Conclusions

The present study was able to demonstrate the association between PA level (expressed according to the PAQ-C score) and the scores of some fitness tests (i.e., handgrip strength; leg stance, sit-and-reach, shuttle-run, long-jump, CMJ peak force, and 20 m sprint) in Italian children aged 8–10 years. Our findings show that, when controlling for gender, age, and BMI, the PAC-Q score is weakly associated with fitness tests such as shuttle-run (i.e., cardiorespiratory fitness), long-jump (i.e., lower-limb muscle power), sprint ability, and sit-and-reach (i.e., low-back flexibility). Conversely, no relationship emerged between PAQ-C, handgrip strength, and CMJ peak force (lower-limb peak force). Boys showed higher values on shuttle-run, sprint, handgrip strength, and long-jump tests than girls. On the contrary, the opposite picture emerged for leg stance, sit-and-reach, and CMJ peak force tests. Older children showed higher outcomes on all proposed physical tests than younger ones. Finally, children’s BMI was positively associated with handgrip strength and CMJ peak force, whereas the opposite trend emerged with shuttle-run, sprint, leg stance, and long-jump tests. Therefore, the “take-home message” of the present study is that no absolute relationship between PA (i.e., PAQ-C score) and the considered fitness tests emerged, and it depends on the test type and children’s characteristics.

## Figures and Tables

**Table 1:** Descriptive data (means and 95% CI) of the subjects’ characteristics, physical activity (i.e., PAQ-C scores), and fitness tests.

	Overall (*n* = 329)	Gender	
		Male (*n* = 174)	Female (*n* = 155)
Age (years)	9.4 (9.3–9.4)	9.4 (9.3–9.5)	9.4 (9.3–9.5)
BMI (kg m^−1^)	18.2 (17.8–18.5)	18.5 (18.1–19.0)	17.8 (17.3–18.3)
PAQ-C (AU)	2.80 (2.09–3.51)	2.82 (1.83–3.82)	2.78 (2.67–2.88)
Handgrip (kg)	15.7 (15.2–16.2)	16.5 (15.8–17.3)	14.7 (14.2–15.3)
Leg Stance (s)	42.1 (38.2–46.0)	38.6 (33.2–44.0)	46.0 (40.3–51.7)
Sit-and-reach (cm)	22 (21–23)	20 (19 -21)	24 (23–25)
Shuttle-run (m)	532 (504–560)	570 (528–613)	489 (454–523)
Long-jump (cm)	116 (114–118)	120 (117–123)	112 (109–115)
Peak-Force-CMJ (N)	574.3 (554.7–593.8)	565.2 (537.7–592.6)	583.8 (55.7–611.9)
Sprint (s)	4.31 (4.27–4.35)	4.25 (4.20–4.31)	4.37 (4.31–4.43)

Notes: BMI: Body Mass Index; PAQ-C: Italian version of the Physical Activity Questionnaire for Older Children.

**Table 2:** LMMs statistical results.

	Handgrip		Single-Leg-Stance		Sit-and-Reach		Shuttle-Run		Long-Jump		Peak-Force-CMJ		Sprint	
	B (SE)	Partial R^2^ (95% CI)	B (SE)	Partial R^2^ (95% CI)	B (SE)	Partial R^2^ (95% CI)	B (SE)	Partial R^2^ (95% CI)	B (SE)	Partial R^2^ (95% CI)	B (SE)	Partial R^2^ (95% CI)	B (SE)	Partial R^2^ (95% CI)
PAQ-C	0.25 (0.25)	0.03 (0.00, 0.14)	0.17 (2.30)	0.13 (0.00, 0.21)	1.86 (0.58)	0.14 ** (0.01, 0.21)	66.82 (17.88)	0.08 *** (0.01, 0.14)	6.00 (1.42)	0.13 *** (0.08, 0.22)	−6.82 (12.31)	0.13 (0.00, 0.23)	−0.09 (0.03)	0.10 *** (0.06, 0.20)
Gender	0.96 (0.31)	0.05 ** (0.00, 0.16)	−5.57 (2.76)	0.14 * (0.04, 0.22)	−4.71 (0.67)	0.23 *** (0.11, 0.29)	86.10 (21.32)	0.08 *** (0.01, 0.14)	8.92 (1.71)	0.14 *** (0.09, 0.23)	−2.20 (0.92)	0.14 ** (0.05, 0.24)	−0.13 (0.03)	0.10 *** (0.06, 0.20)
Age	1.61 (0.34)	0.11 *** (0.03, 0.22)	10.27 (3.82)	0.17 ** (0.08, 0.26)	−1.24 (0.95 **)	0.11 ** (0.01, 0.19)	5.57 (2.27)	0.09 * (0.02, 0.15)	5.57 (2.27)	0.11 * (0.07, 0.20)	42.36 (17.87)	0.11 * (0.02, 0.21)	−0.17 (0.04)	0.15 *** (0.11, 0.24)
BMI	0.37 (0.05)	0.15 *** (0.06, 0.25)	−1.76 (0.45)	0.18 *** (0.09, 0.26)	0.08 (0.11)	0.10 (0.00, 0.19)	−35.22 (3.44)	0.28 *** (0.22, 0.33)	−1.82 (0.28)	0.19 *** (0.15, 0.28)	22.46 (2.48)	0.19 *** (0.11, 0.28)	0.04 (0.01)	0.23 *** (0.18, 0.32)

Notes: PAQ-C: Italian version of the Physical Activity Questionnaire for Older Children; BMI: Body Mass Index; CMJ: Countermovement Squat Jump; B, unstandardized regression coefficients; SE, standard error; *, *p* < 0.05; **, *p* < 0.01; ***, *p* < 0.001.

## Data Availability

The data presented in this study are available on request to the corresponding author. The data are not publicly available due to ethical considerations.
